# A processual model for functional analyses of carcinogenesis in the prospective cohort design

**DOI:** 10.1016/j.mehy.2015.07.006

**Published:** 2015-10

**Authors:** Eiliv Lund, Sandra Plancade, Gregory Nuel, Hege Bøvelstad, Jean-Christophe Thalabard

**Affiliations:** a Department of Community Medicine, Faculty of Health Sciences, University of Tromsø, 9037 Tromsø, Norway; b The MIA-Jouy Research Unit, INRA, France; c CNRS, INSMI Stochastics and Biology Group (PSB) LPMA, UPMC, Sorbonne University, France; d Department of Applied Mathematics, MAP5, 45 rue des Saints-Peres, University Paris Descartes, 75006 Paris, France

## Abstract

Traditionally, the prospective design has been chosen for risk factor analyses of lifestyle and cancer using mainly estimation by survival analysis methods. With new technologies, epidemiologists can expand their prospective studies to include functional genomics given either as transcriptomics, mRNA and microRNA, or epigenetics in blood or other biological materials. The novel functional analyses should not be assessed using classical survival analyses since the main goal is not risk estimation, but the analysis of functional genomics as part of the dynamic carcinogenic process over time, i.e., a “processual” approach. In the risk factor model, time to event is analysed as a function of exposure variables known at start of follow-up (fixed covariates) or changing over the follow-up period (time-dependent covariates). In the processual model, transcriptomics or epigenetics is considered as functions of time and exposures. The success of this novel approach depends on the development of new statistical methods with the capacity of describing and analysing the time-dependent curves or trajectories for tens of thousands of genes simultaneously. This approach also focuses on multilevel or integrative analyses introducing novel statistical methods in epidemiology. The processual approach as part of systems epidemiology might represent in a near future an alternative to human in vitro studies using human biological material for understanding the mechanisms and pathways involved in carcinogenesis.

## Introduction

The dynamics of human carcinogenesis is still remarkably unknown, even after intensive research on the cancer genome [1] and through genomewide association studies (GWAS) [2]. Consequently, functional analyses, here defined as transcriptomics and epigenetics, have been advocated as a future research direction of systems epidemiology [3]. Some have even proposed to move studies of carcinogenesis from animals to “the human model” [4]. Thus, the research challenge is to perform human studies investigating the time-dependent carcinogenic process. This would imply the use of prospective cohort studies with suitable biological material for functional analyses [5]. Presently, the prospective design has primarily been used for risk analyses relating different exposures to disease outcome through statistical modelling and estimation. Exposures could be either lifestyle information or genomic information like single nucleotide polymorphisms, but might equally well be transcriptomic or epigenetic data. This risk-related research has traditionally been used for inferring causal relationships [6]. But functional genomics might offer an additional distinct process-related approach that could be termed *processual*. Processual research describes changes over time in functional genomics related to the carcinogenic process – like for instance, changes in oncogenes or tumour suppressor genes. The aim of this article is to delineate the difference between the causal and the processual approaches and to raise some methodological questions generated by this latter, novel approach.

## From risk factor study to functional analysis

Over the last two decades cohort or prospective studies have been considered as the most valid design for estimating risk of diseases in relation to different exposures or lifestyle factors, as they allow for better control of both selection and information biases, particularly, the alleviation of recall bias observed in case-control studies [7]. Usually, exposure information is assessed only at start of follow-up even though some studies have repeated measures of exposure information.

The need for more extensive and higher cost information in the genomic era encouraged a case-control design nested within a cohort, in which the extensive measures are only carried out in the sample of the cases with controls selected from the cohort and matched on pre-determined relevant covariates. This design has often neglected the time of follow-up by using logistic regression models instead of survival analyses [8]. Clearly, all laboratory analyses of functional markers (mRNA, microRNA, methylation) are expensive, whether they are explored by microarray or by deep sequencing technologies.

The introduction of functional genomics into a prospective design as markers of risk can be analysed in the same models as other exposures as proposed in the “meet-in-the-middle” approach [9]. Again, this is a classical risk factor or causal model.

From the causal perspective, a functional marker may be either an intermediate in the disease process or an exposure marker. This distinction is not immediately evident, due to lack of knowledge about the carcinogenic process. If the functional marker is an intermediate in the disease process, then one would assume that the level would change over time as predicted from current models of carcinogenesis. For instance, mRNA or microRNA could easily be considered as markers of the carcinogenic process changing with time. In such a situation, the analytical approach must be reversed since what we want to estimate is the timeline before diagnosis of changes in the functional marker. This leads to a shift in paradigm. In the processual approach, the primary concern is to estimate the time dependency of the marker in relation to the time before cancer diagnosis.

### Hypothesis: processual model

**Fig. 1:**
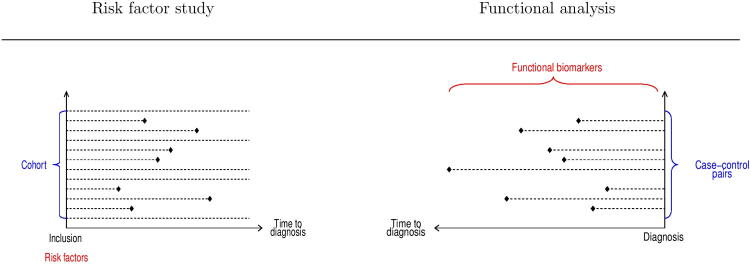
Schematic description of the traditional cohort design for risk estimation (left panel) and the concept of processual analyses within the same framework (right panel).

Consequently, in processual analyses, transcriptomics or epigenetics measured at time of inclusion are considered as measurements at different time points before diagnosis, which is tantamount to a «look backward» in time. This turn of the point of view with respect to risk factor study is illustrated in Fig. 1. The left hand side of Fig. 1 displays a classical prospective GWAS design including genomic data and exposures like use of hormones, smoking or levels of organic pollutants, all measured at the inclusion in the study; the *x*-axis represents the time elapsed since the beginning of the study, and the failure time for a case-control pair corresponds to the diagnosis of the case. The point of view adopted is as follows: given the values of some covariates – genomics and exposures – what is the risk of developing a cancer at some time? Thus, genomics and exposure variables are considered as risk factors for cancer, and the relationship may be expressed in terms of a survival analysis model:

P[T|E,G]

T is the failure time, E the exposures and G the genomics measurements.

The right hand side of Fig. 1 presents a nested case-control design including transcriptomics and exposures measured at inclusion in the study; the *x*-axis represents the time to diagnosis, and for each case-control pair, the time interval between the transcriptomic measurements and diagnosis is displayed.

The analysis of transcriptomics instead of genomics outputs raises a different question: how are transcriptomics data affected by the carcinogenic process? Therefore, transcriptomics are analysed as potential biomarkers of the carcinogenic process and the statistical quantity of interest is the distribution of the gene expression GE as a function of the time to diagnosis T and the exposures E:

P[GE|T,E]

## Discussion

For practical and economic reasons, only a single measurement at time of inclusion may be available for each individual. In this case, the main assumption is that the transcriptome measurements collected on distinct individuals at different times before diagnosis are consequences of the same carcinogenic process. This point of view is commonly adopted in lab experiments, e.g., when dissections performed at different time points on different animals are analysed as a longitudinal study [10]. In an epidemiological context, the individual variability is expected to be much higher due to the heterogeneity of cancer. This approach thus relies on the assumption that available information on the outcome allows stratification according to different biological processes, such as positive or negative node status at time of diagnosis.

Apart from being canonical in prospective nested case-control studies, a survival analysis model may appear appropriate for analysing the links between covariates and time to cancer diagnosis. More precisely, we will discuss the relevance of classical semi-parametric models with either additive or multiplicative effects of the covariates, mostly used in the context of GWAS analysis, where the dimension of the covariate matrix exceeds the sample size. Estimation of additive effects in a survival analysis model requires the knowledge of the covariates at any time before diagnosis [11]. In the Cox model [12] which represents one of the most widely used multiplicative model in epidemiology, the instantaneous risk of occurrence of disease is modelled as a function of the covariates known at time of inclusion. Thus, semi-parametric models with time-varying covariates cannot be estimated from a prospective design including a unique measure at time of inclusion, unless covariates are assumed to be constant over time. Consequently, this assumption would not allow us to address changes in gene expression over time.

### A novel point of view in the epidemiological context

Whereas survival analysis targets exposures possibly associated with cancer, the analysis of transcriptomics and epigenetics requires the inclusion of exposures which may affect gene expression regardless of their carcinogenic effect, in order to improve sensitivity by reducing individual variability. In a purely mechanistic approach for pathway analyses, most studies only concern cases. Information on functional markers is then usually taken from tissues and sometimes from blood at time of diagnosis. Collection of exposure information will thus be liable to recall or information bias. Studies of the cancer genome used for describing the carcinogenic process [1] are unable to identify mutations or functional changes related to exposures. Since different exposures give different phenotypes of cancer, the sensitivity of the analyses could be reduced. Classical examples are the differences in risk estimates of several lifestyle factors and different types of hormone receptor status in breast cancer [13]. On the other hand, controls are necessary as a reference level for functional markers. Without a reference, changes in a functional marker upwards could be either from a low level to normal, or from normal to a high level. In addition, information from controls enables the adjustment of confounding effects of gender, age and lifestyle. The removal of laboratory batch effects in most designs depends on a strict matching through all laboratory procedures. It should be noted that over time controls may become cases and the case-control pair would be removed. If this information is not available through updated register data or clinical information, then one could estimate the proportion from the cohort based incidence rates.

The introduction of functional genomics into the traditional cohort study enables mechanistic analyses of pathways in what has been called a “human model” [4]. The novel processual model depends on mathematical models estimating the changes in the functional markers over time. In our functional approach, we use measurements of gene expression at different time points to estimate the unknown time-dependent function of gene expression. In this situation transcriptomics or epigenetics become markers of the underlying disease process like carcinogenesis and not simply markers of biological risk. The consequences for the use of different statistical methods are not addressed here, but the methods used in integrative clinical cancer research demonstrate the wide range of potential statistical models to be used [14]. Several existing models could be used, especially methods developed for dynamic longitudinal analyses. In risk estimation with the Cox proportional hazard model, time can be considered as a nuisance variable. Commonly used estimators of risk, like relative risk or the proportional hazard, have no time dimension. Researchers are not usually interested in the time distribution, although its values affect the distribution of the observations.

A major challenge to the processual approach is the ability to discriminate between functional changes due to exposures and the disease specific changes due to the inherent disease process [15]. As an example, smoking results in a lot of gene expression signals in blood, but not all of these are related to carcinogenesis.

A last comment should be on the complexity of processual analyses compared to risk estimation. In GWAS analyses with hundreds of thousands of single nucleotide polymorphisms, which are all treated equally in the same statistical model, a major problem is the false discovery rate [16]. In processual research, using mRNA with around 20,000 genes we have no knowledge about the time-dependency of the expression of individual genes and only fragmental knowledge about the complex regulatory interplay between mRNA and up to 1000 microRNA, not forgetting the 450,000 methylation sites, as indicated by the current technology. It emphasises the need for multi-level analyses. It is also unclear to what extent these different functional levels are related to either the exposures or the carcinogenic process or both. Epigenetics could be a marker of chronic exposures while gene expression could be related to ongoing functional changes, like the direct effect of oncogenes or tumour suppressor genes.

## Summary

Our aim has been to describe the need for a change in mathematical models generated by the novel options for transcriptomic analyses in prospective studies as part of a processual approach. The dynamics of carcinogenesis can now be unravelled in a human observational design, thus adding an independent source of knowledge about pathways.

## Conflict of interest

None declared.

## Authors’ contribution

EL is PI of the project and responsible for writing the manuscript. SP did the mathematical formulation. GN, HB and J-C T all participated in the discussions over years in the statistical working group of TICE.
